# *APOC3* may not be a predictor of risk of ischemic vascular disease in the Chinese population

**DOI:** 10.12688/f1000research.5676.2

**Published:** 2014-12-22

**Authors:** Liang Tang, Zhi-Peng Cheng, Qing-Yun Wang, Wei Zeng, Hui Liu, Ying-Ying Wu, Bei Hu, Yu Hu

**Affiliations:** 1 Institute of Hematology, Tongji Medical College, Union Hospital, Huazhong University of Science and Technology, Wuhan, 430022, China; 2 Collaborative Innovation Center of Hematology, Union Hospital, Huazhong University of Science and Technology, Wuhan, 430022, China

**Keywords:** ischemic vascular disease, risk prediction, APOC3

## Abstract

The genetic background of ischemic vascular disease is actively being explored. Several studies have shown that inhibition of *APOC3* significantly reduces plasma levels of apolipoprotein C3 and triglycerides. Recently, the TG and HDL Working Group and Jørgensen *et al.* reported that loss-of-function mutations in *APOC3* are associated with decreased triglyceride levels and a reduced risk of ischemic vascular disease in European and African individuals. We performed a replication study in 4470 Chinese participants. The coding regions of *APOC3 *were amplified and re-sequenced. However, only synonymous and intronic variants with no functional consequences were identified. None of the loss-of-function mutations reported in European and African individuals were observed. Therefore, *APOC3* may not be an ideal predictor for risk of ischemic vascular disease in the Chinese population.

## Correspondence

The genetic basis of ischemic vascular disease such as coronary artery disease is actively being explored. Most studies have focused on susceptibility factors contributing to an increased risk ^1^, while only a few studies have identified protective variants conferring reduced risk ^2^. Recently, the TG and HDL Working Group and Jørgensen *et al.* reported that loss-of-function mutations in *APOC3* are associated with decreased triglyceride levels and a reduced risk of ischemic vascular disease in individuals of European and African ancestry ^3, 4^. Approximately 1 in 150 individuals were heterozygous carriers of at least one of the four mutations: R19X, A43T, IVS2+1G→A, and IVS3+1G→T. Heterozygous carriers for these mutations had a significantly lower incidence of ischemic vascular disease as compared to non-carriers (hazard ratio = 0.59). Triglyceride and circulating *APOC3* levels in the carriers were only 61% and 54% of those in non-carriers, respectively. These critical findings prompted us to undertake a replication study in China, where over one million people are affected by cardiovascular diseases each year ( http://www.nhfpc.gov.cn/).

A total of 4470 unrelated Chinese participants were enrolled, including 1488 healthy controls, 1050 patients with ischemic stroke, 628 patients with coronary artery disease, and 1304 patients with venous thrombosis, which could also be exacerbated by effects on the coagulation system resulting from elevated triglyceride levels. The 1488 healthy controls and 1050 patients with ischemic stroke were described in a previous study ^5^. Briefly, healthy individuals did not present any relevant medical history or family history of ischemic vascular disease. Ischemic stroke was confirmed by brain computed tomography (CT) and/or brain magnetic resonance imaging (MRI). The 1304 patients with venous thrombosis were described previously ^6^. Thrombosis was confirmed by objective investigations such as color Doppler ultrasonography and/or CT angiography. Patients with coronary artery disease were enrolled in our hospital from September 2013 to March 2014. Coronary artery disease was validated by angiographic evidence of at least one segment of a major coronary with over 50% organic stenosis. The characteristics of the 628 patients with coronary artery disease are summarized in Table 1. Written informed consent was obtained from all participants, and the study was approved by the Ethics Committee of Union Hospital affiliated with Huazhong University of Science and Technology (Approval number 2013-03-0052).

**Table 1.** Characteristics of the 628 patients with coronary artery disease.

Characteristic	Number	Percentage
Onset age (yr, mean)	61.6 ± 10.8	
<40	11	1.7%
≥40 and <60	256	40.8%
≥60	361	57.5%
Sex		
male	408	65.0%
female	220	35.0%
Coronary artery disease		
angina pectoris	422	67.2%
myocardial infarction	206	32.8%
Family history		
yes	32	5.1%
no	596	94.9%
Current smoker		
yes	157	25.0%
no	471	75.0%
Drinking		
yes	79	12.6%
no	549	87.4%
Hypertension		
yes	425	67.7%
no	203	32.3%
Type 2 diabetes		
yes	175	27.9%
no	453	72.1%
Fasting serum lipid levels		
TC	3.97 ± 0.99 mmol/L	
TG	1.55 ± 1.05 mmol/L	
HDL-C	1.19 ± 0.29 mmol/L	
LDL-C	2.07 ± 0.76 mmol/L	

TC, total cholesterol; TG, triglycerides; HDL-C, high density lipoprotein cholesterol; LDL-C, low density lipoprotein cholesterol.

The diagnosis of myocardial infarction was based on typical chest pain with a duration over 30 min, on electrocardiographic patterns, and on increased creatine kinase MB isoenzyme and troponin I levels. Hypertension is defined as systolic blood pressure ≥ 140 mmHg and/or diastolic blood pressure ≥ 90 mmHg. Type 2 diabetes were clarified using the 1999 WHO criteria, including fasting plasma glucose ≥ 7.0 mmol/L, 2-hour oral glucose tolerance test plasma glucose ≥ 11.1 mmol/L or ongoing therapy for diabetes.

Blood samples were collected into a vacutainer tube containing 0.105 mol/L trisodium citrate and were then centrifuged at 2000 g for 15 minutes. Genomic DNA was isolated using a salt precipitation method and was then used for sequencing. The four exons and the flanking intronic regions of *APOC3* were amplified by PCR and then sequenced on an Applied Biosystems ABI 3730 Genetic Analyzer, as previously described ^3^. The oligonucleotide pairs and annealing temperatures employed in PCR and sequencing are shown in Table 2. In this study, we identified only synonymous and intronic variants, with no functional consequences, and similar genotype distributions across the groups ( Table 3). None of the loss-of-function mutations reported in European and African individuals were observed in the current cohort. Therefore, the genetic background of ischemic vascular disease is highly variable among different ethnic groups, and *APOC3* may not be an ideal predictor of risk of ischemic vascular disease in the Chinese population.

**Table 2.** Primers and annealing temperatures for PCR and sequencing.

Exon	Forward primer (5'-3')	Reverse primer (5'-3')	AT (ºC)	Product size (bp)
1	GCCTTTACTCCAAACACCCC	AGTGCTTCTCCAGGCTTGCT	58	602
2 and 3	CCTTCTGAGAGCCCGTATTAGC	CCGCAGCAGCCTGACAAA	58	646
4	GGGGCATAAACATCTGAGGT	CTACCAGAAGGTGGATAGAGC	58	693

AT, annealing temperature. The accession number of *APOC3* reference sequence in GenBank is NG_008949.1.

**Table 3.** *APOC3* variants identified in the 4470 Chinese participants.

Variables	dbSNP ID	Control group n = 1488	Ischemic stroke n = 1050	Coronary heart disease n = 628	Venous thrombosis n = 1304
Age (yr, mean)		61.2 ± 12.8	62.2 ± 12.3	61.6 ± 10.8	51.7 ± 13.8
Male, n (%)		978 (65.7%)	691 (65.8%)	408 (65.0%)	638 (48.9%)
Variants, n (%) ^b^					
c.10C>A (p.Arg4=)	novel				
CC		1485 (99.8%)	1048 (99.8%)	627 (99.8%)	1301 (99.8%)
CA		3 (0.2%)	2 (0.2%)	1 (0.2%)	3 (0.2%)
P value			0.95	0.84	0.87
c.99G>A (p.Gln33=)	rs200557528				
GG		1481 (99.5%)	1046 (99.6%)	625 (99.5%)	1296 (99.4%)
GA		7 (0.5%)	4 (0.4%)	3 (0.5%)	8 (0.6%)
P value			0.73	0.98	0.61
c.102T>C (p.Gly34=)	rs4520				
TT		655 (44.0%)	456 (43.4%)	278 (44.3%)	582 (44.6%)
TC		641 (43.1%)	449 (42.8%)	272 (43.3%)	566 (43.4%)
CC		192 (12.9%)	145 (13.8%)	78 (12.4%)	156 (12.0%)
P value			0.80	0.95	0.75
c.179+57G>A	rs2070667				
GG		1098 (73.8%)	776 (73.9%)	454 (72.3%)	967 (74.2%)
GA		329 (22.1%)	235 (22.4%)	148 (23.6%)	286 (21.9%)
AA		61 (4.1%)	39 (3.7%)	26 (4.1%)	51 (3.9%)
P value			0.88	0.76	0.96
c.240G>A (p.Lys80=)	novel				
GG		1483 (99.7%)	1047 (99.7%)	626 (99.7%)	1301 (99.8%)
GA		5 (0.3%)	3 (0.3%)	2 (0.3%)	3 (0.2%)
P value			0.82	0.95	0.60
c.*40G>C	rs5128				
GG		763 (51.3%)	535 (51.0%)	328 (52.2%)	665 (51.0%)
GC		562 (37.8%)	394 (37.5%)	240 (38.2%)	494 (37.9%)
CC		163 (10.9%)	121 (11.5%)	60 (9.6%)	145 (11.1%)
P value			0.90	0.64	0.98
c.*71G>T	rs4225				
GG		1006 (67.6%)	703 (66.9%)	416 (66.2%)	880 (67.5%)
GT		414 (27.8%)	304 (29.0%)	180 (28.7%)	368 (28.2%)
TT		68 (4.6%)	43 (4.1%)	32 (5.1%)	56 (4.3%)
P value			0.73	0.79	0.92

dbSNP, single nucleotide polymorphism database of the National Center for Biotechnology Information ( http://www.ncbi.nlm.nih.gov/projects/SNP).

Comparisons between the controls and each case group were assessed with the use of the chi-square test. A two tailed P<0.05 was considered significant.

However, there are some limitations in this study. First, we only analyzed the coding sequence, as in the studies carried out in European and African individuals. Potential effects from large deletions and mutations in regulatory regions of the gene cannot be excluded. Previous studies have revealed two promoter polymorphisms (T-455C and C-482T) that affect the expression of *APOC3.* However, their relationship with coronary artery disease was not observed in a Han Chinese population ^7^. Second, the consequences of the synonymous mutations and the intronic mutations identified here were only predicted by an on-line bioinformatics tool ( http://www.fruitfly.org/seq_tools/splice.html). Third, the triglyceride levels were not available for the patients with venous thrombosis. Thus, the triglyceride levels according to the genotypes were not further analyzed. Nevertheless, considering the relatively large sample size, we suggest that functional variants in *APOC3* could be very rare in China. Further studies are warranted to understand the genetic basis governing triglyceride levels and conferring protective effects on ischemic vascular disease in the Chinese population.

## Consent

Written informed consent to publish these data has been obtained by each participant.
