# Supplementary material for: APOC3 may not be a predictor of risk of ischemic vascular disease in the Chinese population
Source: F1000Res. 2014 Dec 22;3:270. Originally published 2014 Nov 7. [Version 2] doi: 10.12688/f1000research.5676.2 (PMC4304213; doi:10.12688/f1000research.5676.2)
Supplement: Peer review history

# Peer review: Referee response for version 2

**DOI:** 10.5256/f1000research.6399.r7130

**Published:** 2014-12-23

**Authors:** Bin Zhang

**Affiliations:** 1 Genomic Medicine Institute, Lerner Research Institute of Cleveland Clinic, Cleveland, OH, USA

Authors addressed the main concerns. No further comments.

I have read this submission. I believe that I have an appropriate level of expertise to confirm that it is of an acceptable scientific standard.

# Peer review: Referee response for version 1

**DOI:** 10.5256/f1000research.6066.r6866

**Published:** 2014-12-10

**Authors:** Bin Zhang

**Affiliations:** 1 Genomic Medicine Institute, Lerner Research Institute of Cleveland Clinic, Cleveland, OH, USA

The authors performed a confirmatory study for the recently reported protective effects of loss-of-function mutations in *APOC3* in the Chinese population. Not a single loss-of-function mutation was identified in exons and exon-intron junctions of *APOC3* in a total of 4470 study subjects. The authors concluded that *APOC3* is not a good predictor of risk of ischemic vascular disease in the Chinese population.

This is a worthwhile study and the information, although negative, is certainly useful. However, based on the data reported in the paper, it is premature to conclude that functional variants in *APOC3* are very rare and/or not related to ischemic vascular disease in the Chinese population. The data merely conclude that detrimental mutations are very rare in the sequenced regions of *APOC3* gene in the Chinese population. Potential effects from large deletions of the gene and mutations in regulatory regions of the gene cannot be excluded. In fact, there have been reports of promoter polymorphisms that affect the expression of *APOC3* (ref. 28-31 in Jørgensen et. al). Unless additional, more comprehensive mutational studies are performed in the cohort, conclusions should be modified to reflect the limitations of the current study.

There have been examples of synonymous mutations that affect the translation of mRNA and therefore affect the protein level. Also, intronic mutations distant from splice sites could affect splicing. Authors should clearly state reasons why they believe that the variants identified in the study have no functional consequences.

References should be added to the end of the second sentence of the paper (while only “a” few studies have identified protective variants conferring reduced risk).

I have read this submission. I believe that I have an appropriate level of expertise to confirm that it is of an acceptable scientific standard, however I have significant reservations, as outlined above.

# Peer review: Referee response for version 1

**DOI:** 10.5256/f1000research.6066.r6920

**Published:** 2014-12-05

**Authors:** Chang-Geng Ruan

**Affiliations:** 1 Key Laboratory of Thrombosis and Haemostasis, Ministry of Health, Suzhou, China

Two very recent landmark large-scale studies show that loss-of-function mutations in APOC3 are associated with lower levels of plasma triglycerides, and carriers of these mutations have a reduced risk of coronary heart disease in European and African individuals. Tang *et al*. performed a timely replication study in 4,470 Chinese individuals. Unexpectedly, no loss-of-function mutations identified in the European and African populations were found in the Chinese population. This important study not only highlights the difference in genetic susceptibility to cardiovascular disease in different ethnic populations, but also suggests that APOC3 variants are not applicable to the Chinese population to predict risk for ischemic vascular disease.

APOC3 may still be an important regulator of lipid metabolism in Chinese, and novel variants of this gene remain to be identified in this ethnic population. Consequently, the authors need to change their conclusion from “Therefore, *APOC3* may not be an ideal predictor for risk of ischemic vascular disease in the Chinese population” to “Therefore, *APOC3* variants identified in the European and African population may not be an ideal predictor for risk of ischemic vascular disease in the Chinese population”.

I have read this submission. I believe that I have an appropriate level of expertise to confirm that it is of an acceptable scientific standard.

# Peer review: Referee response for version 1

**DOI:** 10.5256/f1000research.6066.r6688

**Published:** 2014-11-13

**Authors:** Javier Corral

**Affiliations:** 1 Centro Regional de Hemodonación, Universidad de Murcia, Murcia, Spain

Despite being a negative study, the information in this article is valuable. It describes a study that found no relevant mutation in *APOC3* among a large number of Chinese subjects (4470), including 1488 healthy controls, 1050 patients with ischemic stroke, 628 patients with coronary artery disease, and 1304 patients with venous thrombosis. Actually, no one with the loss-of-function mutations reported in European and African individuals was identified in this study. Only synonymous and intronic variants were discovered. The authors indicated that these variants have no functional consequences, but no data are shown. At the least the TG levels according to the genotype might be shown in table 2.

The authors must indicate that other genetic defects, such a gross deletions or regulatory mutations not identified by sequencing methods or by analysis of exons, may cause a loss-of-function of *APOC3* and might be associated with decreased triglyceride levels and a reduced risk of ischemic vascular disease. Actually, selection of subjects with decreased triglyceride levels and a deeper analysis of this gene might be a better strategy to identify these genetic variants potentially involved in TG levels and risk of ischemic vascular disease. Accordingly, I think the authors might change the title of the article.

I have read this submission. I believe that I have an appropriate level of expertise to confirm that it is of an acceptable scientific standard.
